# Construction and Activity Study of a Natural Antibacterial Patch Based on Natural Active Substance-Green Porous Structures

**DOI:** 10.3390/molecules28031319

**Published:** 2023-01-30

**Authors:** Xiangfan Gao, Yuan Zhou, Jinhui Gu, Xinping Liu, Zhijun Zhang

**Affiliations:** 1 Key Laboratory of Surface & Interface Science of Polymer Materials of Zhejiang Province, Department of Chemistry, Zhejiang Sci-Tech University, Hangzhou 310018, China; 2 Department of Pharmacy, Taihe Hospital, Hubei University of Medicine, Shiyan 442000, China; 3 College of Pharmacy, Hubei University of Traditional Chinese Medicine, Wuhan 430065, China; 4 School of Pharmaceutical Science, University of South China, Hengyang 421001, China

**Keywords:** antibacterial patch, antibacterial materials, green products, plant porous structures

## Abstract

Bacterial infections are a serious threat to human health, and the rapid emergence of bacterial resistance caused by the abuse of antibiotics exacerbates the seriousness of this problem. Effectively utilizing natural products to construct new antimicrobial strategies is regarded as a promising way to suppress the rapid development of bacterial resistance. In this paper, we fabricated a new type of natural antibacterial patch by using a natural active substance (allicin) as an antibacterial agent and the porous structure of the white pulp of pomelo peel as a scaffold. The antibacterial activity and mechanisms were systematically investigated by using various technologies, including the bacteriostatic circle, plate counting, fluorescence staining, and a scanning electron microscope. Both gram-positive and negative bacteria can be effectively killed by this patch. Moreover, this natural antibacterial patch also showed significant anti-skin infection activity. This study provides a green approach for constructing efficient antibacterial patches.

## 1. Introduction

Bacterial infection is a serious threat to public health. In our daily life, how to effectively combat skin wound infection is a problem that we often face. The antimicrobial components that are commonly used in anti-wound skin infection strategies include antibiotics [1], antimicrobial peptides [2,3], and silver-containing compound nanomaterials [4,5,6,7]. The long-term use of these drugs, especially antibiotics, easily causes bacterial resistance. In addition, most of these antibacterial components show strong interaction with phospholipid membranes or biomacromolecules, and there are non-negligible cytotoxic side effects. Moreover, the cost may also need to be considered, especially for people in poor countries or regions. Therefore, there is an urgent need to develop new anti-wound infection strategies that are of low cost and without side effects.

Natural photosynthesis provides humans with abundant natural products, including a variety of active substances and matrix materials. For example, garlic, which we often eat, is a natural substance rich in active ingredients (e.g., allicin) [8,9]. Allicin, an organic sulfide compound (allyl-2-aminoethyl sulfonate), has many physiological activities such as antibacterial [10,11,12], anti-inflammatory [13,14], and antioxidant functions [15,16,17]. Allicin interacts with the various sulfhydryl-dependent enzyme systems (cysteine proteases and alcohol dehydrogenases) to disturb the bacterial metabolism [11]. In addition, allicin can cause other harmful effects on bacteria; for example, it can change the permeability of cell membranes through lipid peroxidation to allow intracellular contents overflow [18,19], and it may also inhibit bacterial protein and nucleic acid synthesis [20,21]. These effects can affect the activity and growth of bacteria, and thereby allicin provides a remarkably broad-spectrum of antibacterial activity. The antibacterial mechanism is completely different from other antibiotics, which makes it 1000 times less easy to develop resistance compared with other antibiotics [22]. Apart from the unique antibacterial properties, allicin has poor stability when exposed to the atmosphere; therefore, it is generally recommended to use a special scaffold matrix to adsorb allicin for activity maintenance [23,24]. In addition to active substances, many natural products can be used as important matrix materials. For example, the white pulp of pomelo peel, a porous soft biomass with abundant polysaccharides (pectin, cellulose, hemicellulose) and other valuable compounds, is an ideal natural material for dressing. Pomelo peel also has the advantages of low price, easy availability, and it is naturally degradable. Owing to these positive properties, the white pulp of pomelo peel has been widely used as a raw material in various fields [25]. For instance, due to the porous structure and abundant functional groups (e.g., hydroxyl, carbonyl, carboxyl and phenolic groups), pomelo peels have been frequently utilized as an adsorbent for wastewater treatment [25]. Carbonized pomelo peel was found to be a promising and sustainable anode for high energy density asymmetric supercapacitors [26]. Moreover, the carbonized pomelo peel can also be used as a porous scaffold to fabricate efficient catalysts, [27] and even as photothermal materials for solar energy harvest [28]. Nevertheless, the application of pomelo peel in the biomedical field is rarely reported, and the using of pomelo peel as a scaffold for medical dressing has yet to be reported.

**Scheme 1:**
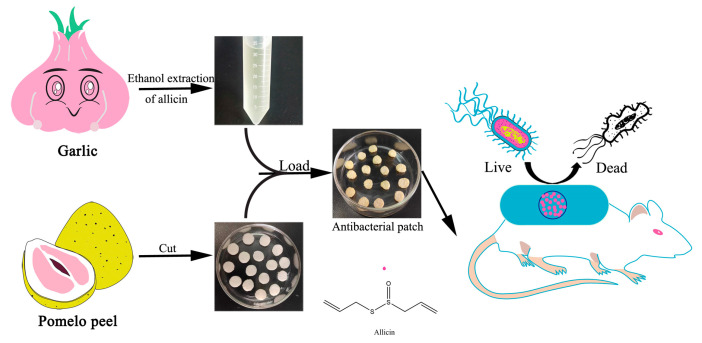
Schematic illustration of the preparation of the natural antibacterial patches.

Inspired by the promising prospect that making full use of natural products is an effective way to construct new strategies against wound infection, in this work, a natural antibacterial patch was prepared with a natural active compound as an antibacterial agent and pomelo peel porous structures as the scaffold (Scheme 1) [29]. Through a series of experiments such as scanning electron microscopy, antibacterial experiments, and Live/Dead staining, the antibacterial patches were demonstrated to have good antibacterial properties and biocompatibility, which can even be used for wound infection treatment.

## 2. Results and Discussion

### 2.1. Extraction of Allicin

**Figure 1:**
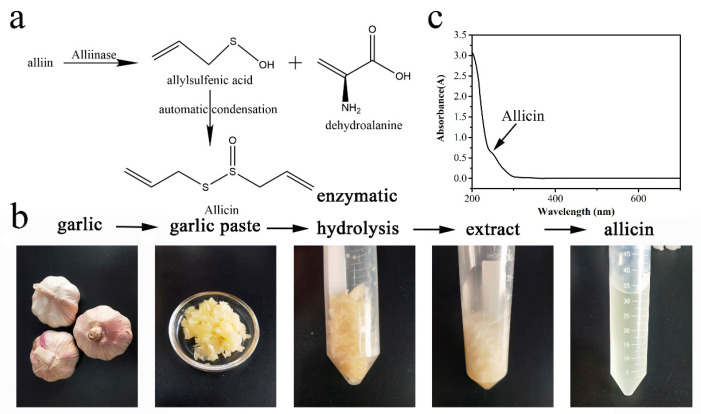
(**a**) Schematic illustration of allicin synthesis mechanism, (**b**) photographs of the allicin extraction process, (**c**) UV-vis spectrum of allicin in ethanol.

The synthesis of allicin is an inherent defense strategy of garlic. Briefly, when garlic is physically damaged, alliinase in garlic cleaves alliin into allyl sulfonic acid and dehydroalanine, then two molecules of allyl sulfonic acid automatically dehydrate and condense to form allicin (Figure 1a) [30,31,32,33,34]. Inspired by this natural synthesis process, we grind garlic into a paste to let it enzymatically hydrolyze (Figure 1b). In view of the hydrophobic property of allicin, after the enzymatic hydrolysis, ethanol was added to extract allicin. The extract showed a typical characteristic absorption peak of allicin around 254 nm (Figure 1c), indicating the successful preparation of allicin. Considering that the hydrolysis time and temperature, the extract time and temperature, and the liquid-solid ratio may influence the final concentration of allicin, we further investigated the extract of allicin under these different conditions. Allicin content in the extract was measured by using L-cysteine as a standard substance and 5, 5′-dithiobis 2-nitrobenzoic acid (DTNB) as an indicator (Supplementary Material Figures S1 and S2). The condition of 30 min of enzymolysis at 30 °C, a liquid-solid ratio (m/m) of 2, and extraction at 30 °C for 90 min was found to be the optimal condition for allicin extraction (Supplementary Material, Figure S3). At the optimum condition, the allicin concentration is around 9.80 mM, which is comparable or better than that of most reported allicin extraction methods [35,36].

### 2.2. Preparation of Antibacterial Patches

**Figure 2:**
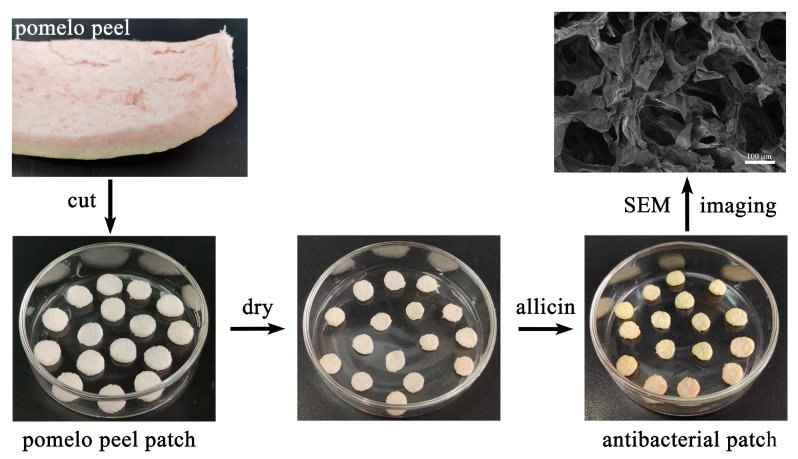
The antibacterial patch preparation processes by using pomelo peel as the scaffold and the extracted allicin as the antibacterial agent.

As shown in Figure 2, pomelo peel was used as the scaffold, and allicin was used as an antibacterial agent for antibacterial patch preparation. The pomelo peel is rich in polysaccharide and has a porous structure, which is an ideal natural material for dressing. Through simple cutting, drying, and allicin adsorption, the antibacterial patches can be obtained in a considerable amount. A scanning electron microscopy (SEM) image shows that the patch has a uniformly distributed porous structure. The pore size of the patch is tens of microns and is formed by the stacking of fold sheet structures. The liquid adsorption capacity was further measured. As shown in Supplementary Material, Figure S4, the patches possess strong water absorption ability, which can absorb nine times the weight of water than its own mass. Hence, the porous structure and liquid absorption ability can facilitate tissue fluid absorption during the antibacterial process.

### 2.3. Antibacterial Activity

#### 2.3.1. Bacteriostatic Ring Test

**Figure 3:**
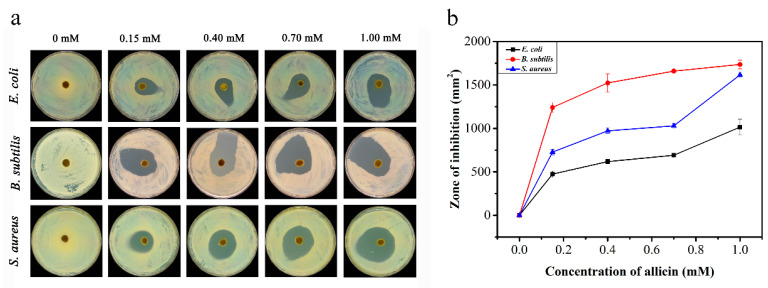
(**a**) Photographs of the inhibition zone after adding different concentrations (0 mM, 0.15 mM, 0.40 mM, 0.70 mM, 1.00 mM) of allicin to pomelo peel. (**b**) The area of the inhibition zone corresponds to Figure (**a**).

In the bacteriostatic ring test, the inhibition zone is often measured to evaluate the antibacterial effect of the material, and a larger zone area means stronger antibacterial activity. As shown in Figure 3a, the pomelo peel patch without allicin showed no obvious inhibition zone, indicating that there is no antibacterial activity. However, the patches that adsorbed with allicin showed an unmistakable suppression zone which was enlarged with the increase of the allicin concentration. These results clearly demonstrated the significant antibacterial activity of the prepared antibacterial patches for *Escherichia coli* (*E. coli*), *Bacillus subtilis* (*B. subtilis*) and *Staphyloccocus aureus* (*S. aureus*), which is attributed to the release of allicin from the patch. The inhibition zone areas were further measured and are shown in Figure 3b. The results displayed that the antibacterial patches have the strongest antibacterial effect against *B. subtilis* and the weakest antibacterial activity against *E. coli*, implying that the antibacterial patches have a stronger antibacterial effect on gram-positive bacteria.

#### 2.3.2. Plate Counting Method

**Figure 4:**
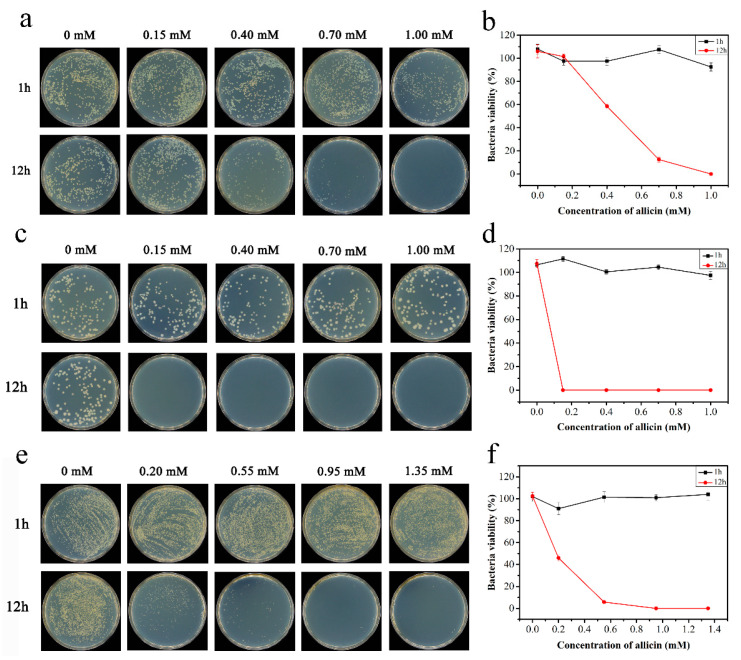
Photographs of bacterial colonies of *E. coli* (**a**), *B. subtilis* (**c**) and *S. aureus* (**e**) treated by the antibacterial patches with different concentrations of allicin. (**b**), (**d**) and (**f**) show the corresponding colony number in (**a**), (**c**) and (**e**), respectively.

In order to further quantitatively evaluate the antibacterial activity of the antibacterial patches, the plate counting-based antibacterial test was then carried out. As shown in Figure 4, the antibacterial patches didn’t show an obvious inhibition effect in a short time for the bacteria (*E. coli*, *B. subtilis* and *S. aureus*). However, a strong antibacterial effect was observed when elongating the incubation time from 1 to 12 h. Meanwhile, the counting results indicated that allicin exhibits an excellent suppressing effect for three bacteria, of which the inhibition efficiency for *B. subtilis* is highest (Figure 4b, d, f). The IC_90_ (concentration that induces a 90% decrease of the colonies) of the antibacterial patch (allicin) on *B. subtilis*, *S. aureus*, and *E. coli* was around 0.15 mM, 0.5 mM and 0.7 mM, respectively. These results indicated that the gram-positive bacteria growth was more affected by the antibacterial patch compared to gram-negative bacteria, which is consistent with the antibacterial effect of allicin on gram-negative and positive bacteria [37]. In addition, in a short time incubation (e.g. 1 h), the antibacterial patch showed no obvious antibacterial effect on gram-positive bacteria and gram-negative bacteria, which may be related to the release rate of allicin from the scaffold. In terms of the timeliness requirements of antibacterial therapy, this is a deficiency of the antibacterial patch which should be improved.

### 2.4. Live/Dead Staining

**Figure 5:**
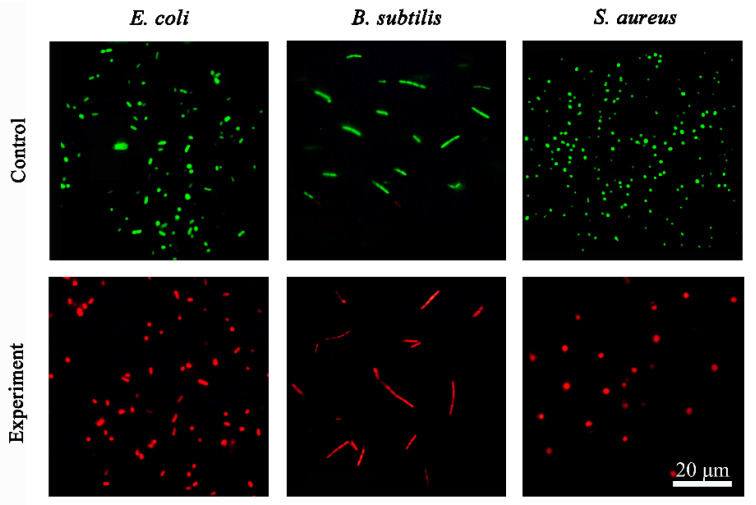
Fluorescence images of the bacteria (*E. coli*, *B. subtilis*, *S. aureus*) stained with SYTO 9 and PI. Scale bar 20 μm.

Live/Dead staining was carried out with a bacterial viability kit containing SYTO 9 and propidium iodide (PI) to study the antibacterial mechanism. SYTO 9 and PI are both nucleic acid-binding dyes. SYTO 9 can permeate the membrane of live cells to give a green fluorescence, and PI can only penetrate the damaged cell membrane to give a red fluorescence. As shown in Figure 5, untreated bacteria (*E. coli*, *B. subtilis* and *S. aureus*) showed strong green fluorescence, while the antibacterial patches treated with bacteria showed a strong red fluorescence, indicating the induced influence of the bacterial membrane permeability by the patches.

### 2.5. Bacterial Morphological Changes

**Figure 6:**
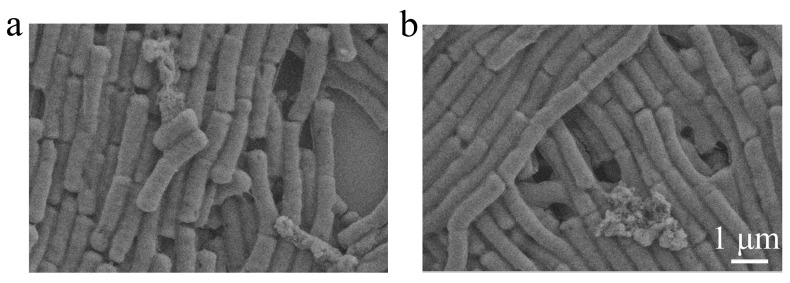
SEM of *B. subtilis* before (**a**) and after (**b**) being treated with an antibacterial patch.

The morphology change of the bacteria after antibacterial patch treatment was further studied by using *B. subtilis* as a model stain. As shown in Figure 6, both the bacteria before and after being treated showed smooth surfaces and integrated structures. This result indicated that the antibacterial patch cannot cause serious damage to the bacterial structures. Combined with the results of Live/Dead staining, we speculate that the changes of the bacterial membrane permeability and the effect on the internal physiological system of bacteria by allicin may be the main reason for the antibacterial patch.

### 2.6. Cytotoxicity Study

**Figure 7:**
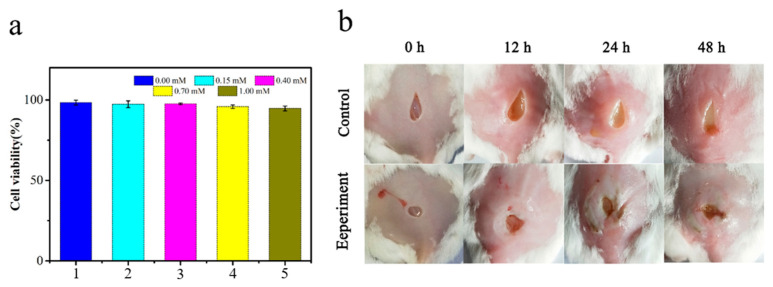
(**a**) Cytotoxic potential of allicin at different concentrations (1 = 0 mM, 2 = 0.15 mM, 3 = 0.40 mM, 4 = 0.70 mM, 5 = 1.00 mM) on A549 cells. (**b**) Photographs of mice wound 0 h, 12 h, 24 h, 48 h after bacterial infection.

In addition, the biocompatibility of antibacterial pomelo peel was investigated by the MTT method. As shown in Figure 7a, the cell viability was still as high as 96% after incubation with the pomelo peel with a high concentration of 1.00 mM allicin. This indicates that allicin in a dose-dependent manner had inhibitory effects on three bacteria (*E. coli*, *B. subtilis* and *S. aureus*), but exhibited no cytotoxicity to A549 cells. As we all know, a cell survival rate of more than 75% can be regarded as non-cytotoxic [38], so the antibacterial pomelo peel which was selected in this experiment has good biocompatibility.

### 2.7. Therapeutic Effect against Mouse Wound Infection

We eventually fabricated a mouse skin wound infection model to test the practical performance of the natural antibacterial patch. As shown in Figure 7b, the mouse skin wounds infected by *B. subtilis* showed obvious suppuration symptoms. However, in the antibacterial patch treatment group, the wounds’ purulent symptoms were much lighter, and scabbing even occurred two days later, which demonstrated the practical performance of the natural antibacterial patch for wound infection treatment.

## 3. Materials and Methods

### 3.1. Materials and Measurement

Purple garlic (purchased from the local market), anhydrous ethanol, DTNB, L-cysteine, 3-(4,5-Dimethylthiazol-2-yl)-2, 5-Diphenyltetrazolium Bromide (MTT) was provided by Aldrich Chemical Co. (Shanghai, China); Dimethyl sulfoxide (DMSO) and UV-vis adsorption spectra were measured by a UV-1900 spectrometer (Shimadzu Corporation, Kyoto, Japan). Scanning electron microscopy (SEM) was conducted using a JSM-5610LV (Japan Electron Optics Laboratory Co., Ltd (JEOL), Kenji Kazato, Tokyo, Japan), and the brightfield and fluorescence images were taken under a Nexcope NIB900-FL fluorescent microscope with Nexcan-T6CCD digital camera (Ningbo Yongxin Optics Co.,Ltd, Ningbo, China). Two dyes, SYTO 9 and PI, came from a bacterial activity kit from Thermos Fisher Scientific (Waltham, MA, USA) and were used for Live/Dead staining. Cell proliferation took place in a DPH-9042CO_2_ incubator (Crystal Technology & Industries, Inc., Addison, TX, USA). The number of bacteria was obtained by an ICount 11 automatic Colony Counter (Shineso Technology Co., Ltd. Hangzhou, China). Bacteria-coated plates were operated in a BHC-800IIB2 biological safety cabinet (Qingdao Haier Biomedical Co., Ltd, Qingdao, China).

### 3.2. Allicin Extract

Fresh purple garlic bulbs (*Allium sativum* L.) were purchased from the Qiantang District Food Market, Hangzhou City, Zhejiang Province, China. The washed garlic bulbs were ground into a paste and placed in an oven at a certain temperature for enzymatic hydrolysis. Ethanol was then added to extract allicin. The supernatant was collected by centrifuge treatment. In this experiment, in order to obtain the highest content of allicin in the selected garlic, the following five conditions were set: Different extraction times, different liquid-solid ratios, different enzymatic hydrolysis temperatures, different enzymatic hydrolysis times, and different extraction temperatures. The condition of 30 min of enzymolysis at 30 °C, a liquid-solid ratio (m/m) of 2, and extraction at 30 °C for 90 min were the optimum conditions for allicin extraction.

### 3.3. Quantitative Determination of Allicin

Allicin content in the extract was measured by using L-cysteine as a standard substance and DTNB (5, 5′-dithiobis 2-nitrobenzoic acid) as an indicator. The detection mechanism was illustrated in Supplementary Material, Figure S1. Briefly, one molecule of allicin reacts with two molecules of L-cysteine to generate two molecules of S-AMC (C_3_H_5_-S-S-CH_2_CH(NH)_2_COOH). The excess L-cysteine reacts with DTNB (5, 5′-dithiobis 2-nitrobenzoic acid) to generate a yellow product, NTB (2-nitro-5-thiobenzoic acid), with a maximum absorbance at 412 nm (Supplementary Material, Figure S2). The absorption peak was detected to evaluate the amount of L-cysteine reacted with DTNB. Subsequently, the content of the allicin was obtained according to Equation (1).

(1) $$C_{allicin}\left(\frac{mmol}{mL}\right)=\frac{\Delta A_{412}\;\times \;d}{2\;\times \;14,150}\;\Delta A_{412}={\;A}_{0}-A\;$$

where A_0_ is the absorbance obtained from the reaction between DTNB and L-cysteine before the reaction; d is the total dilution factor; and 14,150 is the molar extinction coefficient of NTB at 412 nm (1 cm optical path) [39].

### 3.4. Preparation of Pomelo Peel Patches

Fresh pomelo was purchased from a local supermarket. The white pulp in the middle layer was collected and cut into even sheets. The uniform patches with a diameter of 10 mm were obtained by a puncher. The patches were dried in an oven at 30 °C. Finally, allicin with different concentrations was dropped on the patches and dried at room temperature for further use.

### 3.5. Water Absorption of Pomelo Peel

After soaking in deionized water for different times at room temperature, round slices of pomelo peel with a diameter of 10 mm were taken out and suspended for 1 min, and the weight was measured immediately. Water absorption (Q) is defined as:

(2) $$Q\;=\frac{M_{1}-M_{0}}{M_{0}}\times 100\%$$

where M_0_ and M_1_ represent the weight of patches before and after soaking in water, respectively [38].

### 3.6. Antibacterial Activity

All of the following antibacterial experiments were carried out in secondary biological safety cabinets, and UV lamps were turned on for sterilization and disinfection for 15 min before and after each experiment.

#### Bacterial Culture Medium Configuration

Liquid medium configuration: Add 0.5 g yeast extract, 1 g tryptone, 1 g sodium chloride per 200 mL deionized water; solid medium configuration: Add 0.5 g yeast extract, 1 g tryptone, 1 g per 100 mL deionized water Sodium chloride, about 1.5 g of agar powder (liquid media with 3.0% agar). After the medium configuration is completed, put it into an autoclave for sterilization for about 40 min. In this experiment, *E. coli* (ATCC 25922) was selected as Gram-negative bacteria, and *B. subtilis* (ATCC 6051) and *S. aureus* (ATCC 25923) were selected as Gram-positive bacteria model strains. 50 μL of bacterial solution from the original strain solution was added into 2 mL of lysogeny broth (LB) medium, shaken for 8 h with a shaker at a frequency of 180 r/min at 37 °C, and, finally, the OD value at 600 nm of the bacterial solution was measured to be 1.5 for subsequent experiments [40].

1. Agar plating method

40 μL of bacterial solution from the above bacterial liquid with an OD_600nm_ of 1.5 was placed dropwise into a plate containing a solid medium. Evenly spread the bacteria along the inner wall of the plate with a bacterial coating rod. The pomelo peel with different contents of allicin was slowly placed in the center of the plate covering with bacteria with tweezers. All LB-agar plates were kept under aseptic conditions at 37 °C for 12 h.

2. Plate counting method

Allicin with different concentrations (0 mM, 0.15 mM, 0.40 mM, 0.70 mM, 1.00 mM) was dropwise into the pomelo peel and incubated for 12 h after diluting the above bacterial solution with an OD_600nm_ of 1.5 by a certain number of times. 30 μL from the bacterial solution were placed dropwise into a plate containing a solid medium. Evenly spread the bacteria along the inner wall of the plate with a bacterial coating rod. All LB-agar plates were kept under aseptic conditions at 37 °C. *E. coli* and *B. subtilis* were cultured for 12 h and *S. aureus* for 18 h. The control group was incubated without pomelo peel.

### 3.7. Live/Dead Staining

A Live/Dead bacterial activity kit (BacLight^TM^, Thermo Fisher Scientific, Waltham, MA USA) was used to evaluate the bacterial viability [41]. This kit contains two dyes, SYTO 9 and propidium iodide (PI). SYTO 9 can penetrate the cell membrane of live bacteria and show green fluorescence under blue light excitation, while PI can only penetrate the cell membrane of dead cells and show red or orange fluorescence. Bacteria (optical density at 600 nm is 0.5) were incubated with the antibacterial patch for 12 h at room temperature. After washing the bacteria twice with 0.85% NaCl solution, SYTO 9/PI dyes were added and incubated for 20 min in the dark. After this, the bacteria were washed and imaged under a fluorescence microscope. *E. coli*, *B. subtilis* and *S. aureus* without any treatment were used as the control group.

### 3.8. Morphology of Bacteria

500 μL of *B. subtilis* (OD_600nm_ = 1.0) in 0.85% NaCl was incubated with the antibacterial patches (allicin concentration is 1 mM) at room temperature overnight. The bacterial suspension was then collected by centrifuge, and fixed with glutaraldehyde with a volume fraction of 2.5% at room temperature for 4 h [40]. A volume fraction of 20%, 40%, 60%, 80%, and 100% ethanol gradient dehydration was then added. The last of the bacteria were suspended in 100% ethanol solution, and 20 μL was dropped into the silicon wafer with a pipette gun, and the morphological changes were observed under a scanning electron microscope by spraying gold.

### 3.9. Cytotoxicity Study

The cytotoxicity of allicin-adsorbed pomelo peel on human lung adenocarcinoma A549 cells was detected by the MTT method. A total of 100 μL of high-concentration cell suspension was added into the 96-well plate, removing the original medium after the cells are completely adherent. Add the freshly prepared complete medium (DMEM containing 10% fetal bovine serum and 1% penicillin-streptomycin) high-glucose medium), and then pour allicin with different concentrations into pomelo peel (0 mM, 0.15 mM, 0.40 mM, 0.70 mM and 1.00 mM) incubated for 12 h in a CO_2_ incubator. Then aspirate the original medium, wash 2–3 times with PBS (pH = 7.4), add 20 μL MTT (5 mg mL^−1^), put it into the incubator for 4 h, and remove the medium and add 150 μL DMSO solution. Shake for 10 min on a shaker to fully dissolve it, and measure the absorbance of each well of the 96 well plates at OD_490nm_ of the microplate reader. Cells without material were selected as the control group.

### 3.10. In Vivo Antibacterial Experiments

In order to evaluate the antibacterial properties of the allicin-adsorbed pomelo peel in vivo, Kunming mice were selected as the back wound model in this experiment, which was approved by the Ethics Committee of Animal Experiments in Zhejiang Sci-Tech University, and all procedures followed the guidelines for animal experiments in Zhejiang Sci-Tech University. Adult mice were selected as experimental subjects, and the long hairs on the skin surface of the back of the mice were shaved with a shaver, and then the remaining fine hairs were completely removed with a depilatory cream (Supplementary Material, Figure S5). Under ether anesthesia, the epidermis of about 3 mm was cut off with scissors, and 10 μL (OD_600nm_ = 1.5) of *B. subtilis* was added to the wound with a pipette. The pomelo peel with a concentration of allicin was attached to the wound as the experimental group. For the first two days, the pomelo peel (allicin content 1.00 mM) was changed every day, and for the next two days the wound was allowed to heal naturally at room temperature to observe the healing.

## 4. Conclusions

We successfully fabricated a useful natural antibacterial patch. The natural active substance (allicin) and the scaffold materials were prepared in a convenient approach. The antibacterial activity was systematically demonstrated by different assays, including the bacteriostatic circle, plate counting, fluorescence staining, and a scanning electron microscope. Both gram-positive and negative bacteria can be effectively killed by this patch. Moreover, this natural antibacterial patch also showed significant anti-skin infection activity. To sum up, this natural antibacterial patch is convenient for preparation and has high antibacterial activity, which is promising for wound infection treatment. This study provides a green approach for constructing efficient antibacterial patches.

## Data Availability

The data are contained within the article.

## Supplementary Materials

The following supporting information can be downloaded at: https://www.mdpi.com/article/10.3390/molecules28031319/s1, Figure S1: NTB is the reaction process of detecting allicin content by the signal substrate; Figure S2: UV-vis absorption of NTB; Figure S3: Optimal conditions for extracting allicin. (a) Different extraction times with extraction temperature was 35 °C, the enzymolysis time was 30 min, the liquid-solid ratio was 5, and the enzymolysis temperature was 35 °C. (b) Different liquid-solid ratios (m/m) with extraction time were 60 min, the extraction temperature was 35 °C, the enzymolysis time was 30 min, and the enzymolysis tem-perature was 35 °C. (c) Different enzymatic hydrolysis temperatures with extraction time were 60 min, the extraction temperature was 35 ℃, the enzymolysis time was 30 min, the liquid-solid ratio was 5, and the enzymolysis temperature was 35 °C. (d) Different enzymatic hydrolysis times with extraction time was 60 min, the extraction temperature was 35 °C, the liquid-solid ratio was 5, and the enzymolysis temperature was 35 °C. (e) Different extraction temperatures with extraction time were 60 min, the enzymolysis time was 30 min, the liquid-solid ratio was 5, and the enzymolysis temperature was 35 °C; Figure S4: Water absorption of the antibacterial patches; Figure S5: Photographs of the process of establishing a mice wound model. (a) Photographs of shaved mice. (b) Photographs of mice injected with B. subtilis. (c) Mice with "Antibacterial patches".

## Sample Availability

Samples of the compounds are available from the authors.
