# Supplementary material for: Construction and Activity Study of a Natural Antibacterial Patch Based on Natural Active Substance-Green Porous Structures
Source: Molecules. 2023 Jan 30;28(3):1319. doi: 10.3390/molecules28031319 (PMC9918939; doi:10.3390/molecules28031319)
Supplement: Supplementary file 1 [file molecules-28-01319-s001.zip › molecules-2131044-supplementary.pdf]

molecules

Supplementary Materials

Construction and Activity Study of a Natural Antibacterial Patch Based on Natural Active Substance-Green Porous Structures

Xiangfan Gao [1,†], Yuan Zhou [2,3,†], Jinhui Gu [1], Xinping Liu [4] and Zhijun Zhang [1,*]

1 Department of Chemistry, Key Laboratory of Surface & Interface Science of Polymer Materials of Zhejiang Province, Zhejiang Sci-Tech University, Hangzhou 310018, China
2 Department of Pharmacy, Taihe Hospital, Hubei University of Medicine, Shiyan 44200, China
3 College of Pharmacy, Hubei University of Traditional Chinese Medicine, Wuhan 430065, China
4 School of Pharmaceutical Science, University of South China, Hengyang 421001, China
* Correspondence: zjzhang@zstu.edu.cn
† These authors contributed equally to this work.

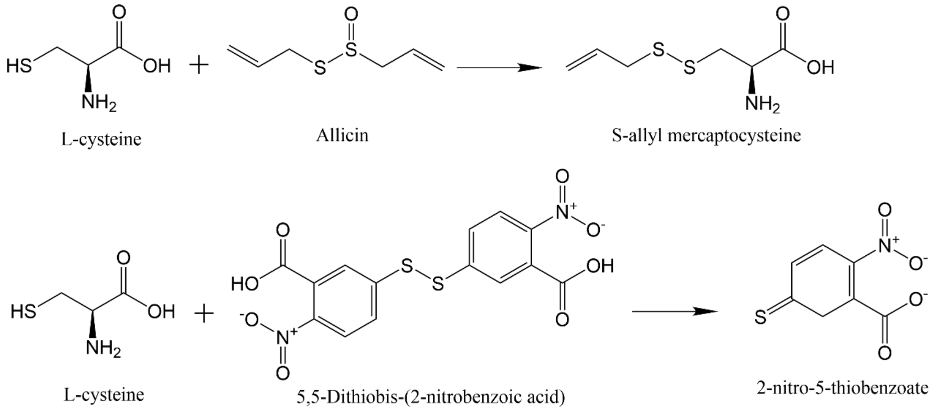

Figure S1. NTB is the reaction process of detecting allicin content by the signal substrate.

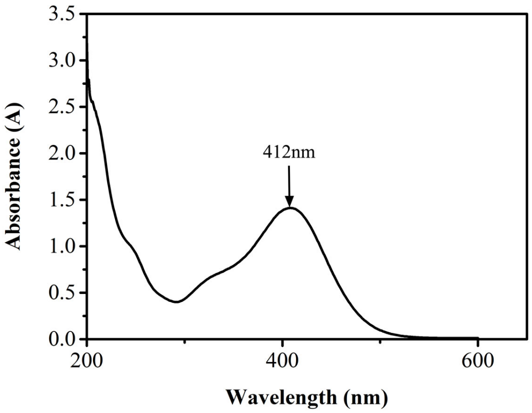

Figure S2. UV-vis absorption of NTB.

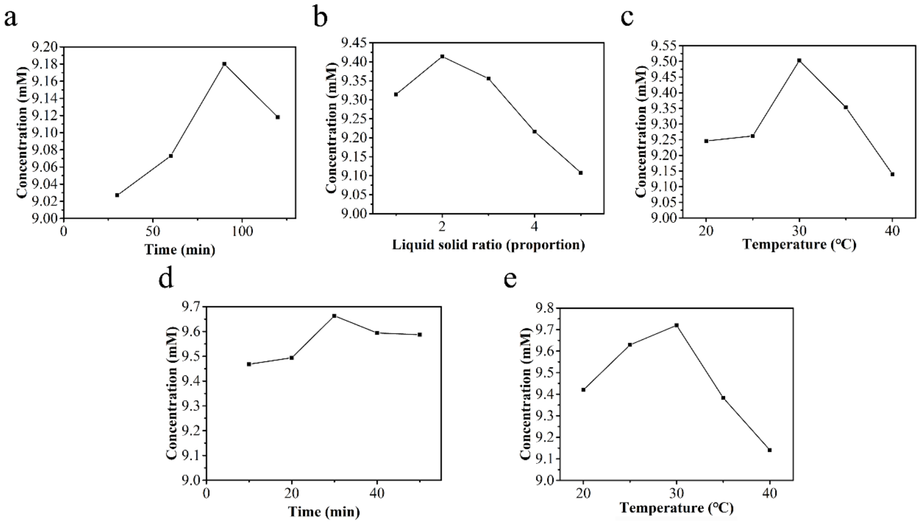

Figure S3. Optimal conditions for extracting allicin. (**a**) Different extraction times with extraction temperature was 35 °C, the enzymolysis time was 30 min, the liquid-solid ratio was 5, and the enzymolysis temperature was 35 °C. (**b**) Different liquid-solid ratios (m/m) with extraction time were 60 min, the extraction temperature was 35 °C, the enzymolysis time was 30 min, and the enzymolysis temperature was 35 °C. (**c**) Different enzymatic hydrolysis temperatures with extraction time were 60 min, the extraction temperature was 35 °C, the enzymolysis time was 30 min, the liquid-solid ratio was 5, and the enzymolysis temperature was 35 °C. (**d**) Different enzymatic hydrolysis times with extraction time was 60 min, the extraction temperature was 35 °C, the liquid-solid ratio was 5, and the enzymolysis temperature was 35 °C. (**e**) Different extraction temperatures with extraction time were 60 min, the enzymolysis time was 30 min, the liquid-solid ratio was 5, and the enzymolysis temperature was 35 °C.

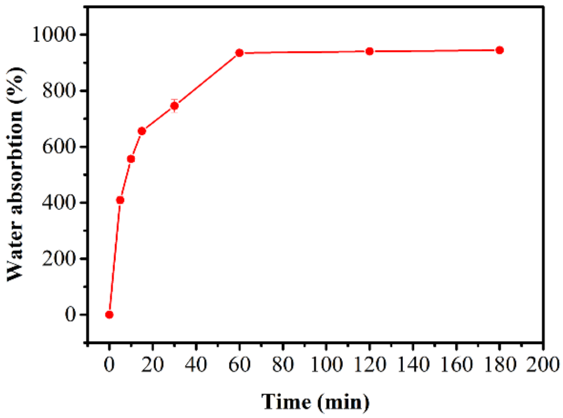

Figure S4. Water absorption of the antibacterial patches.

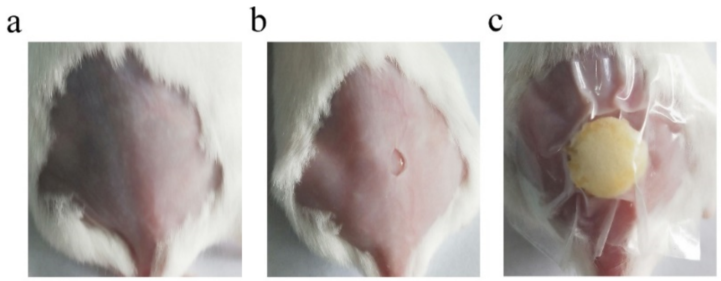

Figure S5. Photographs of the process of establishing a mice wound model. (**a**) Photographs of shaved mice. (**b**) Photographs of mice injected with *B. subtilis.* (**c**) Mice with "Antibacterial patches".
